# Effect of *Melia azedarach* seed mediated nano-ZnO on growth performance, protein utilisation efficiency, haematology and nutritional status in pigs

**DOI:** 10.1007/s11250-024-04217-2

**Published:** 2024-10-31

**Authors:** E. Dinga, U. Marume, G. M. Chelopo

**Affiliations:** 1 Department of Animal Science, School of Agricultural Sciences, Faculty of Agriculture, Science and Technology, North West University, P Bag X 2046, Mmabatho, South Africa; 2 Food Security and Safety Niche Area, Faculty of Agriculture, Science and Technology, North West University, P Bag X 2046, Mmabatho, 2735 South Africa

**Keywords:** *Melia azedarach* seed, ZnO nanoparticles, Growth performance, Protein utilisation efficiency, Haematology

## Abstract

The current study was conducted to investigate the effect of *Melia azedarach* seed-mediated ZnO nanoparticles on growth performance, protein utilisation efficiency, haematology and nutritional status in pigs. A total of 48 pigs were allocated to the following six treatments replicated 8 times: Negative Control (NC, No antibiotic), Treatment 2: Positive control (PC) given a conventional antibiotic (Oxytetracycline, 40 mg/kg feed); Treatment 3: Nano-ZnO 300 mg/L (N300ZnO), Treatment 4: Group given 150 mg/L *Melia azedarach* seed mediated nano-ZnO (N150MA), Treatment 5: Group given 300 mg/L *Melia azedarach* seed mediated nano-ZnO (N300MA), Treatment 6: Group given 450 mg/L *Melia azedarach* seed mediated nano-ZnO (N450MA). The experiment was conducted over 7 weeks. *Melia azedarach* seed-mediated ZnO nanoparticles had no significant effect on growth performance apart from average daily feed intake (ADFI) with treatment 3 having the highest value. It significantly affected protein consumption and growth efficiency but not protein efficiency ratio and specific growth rate. *Melia azedarach* seed-mediated ZnO nanoparticles had no significant impact on nutritional parameters, serum minerals apart from phosphorus which can negatively affect renal functioning.

## Introduction

The global human population has been rapidly increasing over the years, and there is no doubt that the expansion of the pig industry would significantly contribute to resolving the associated food security issues. Nevertheless pigs, young pigs in particular are susceptible to various challenges among them digestive disorders, reduced growth, increased diarrhoea, and higher morbidity and mortality, all of which cause significant economic losses to the pig industry (Smith et al. 2010; Omonijo et al. 2018). Resultantly, antibiotic growth promoters have been used widely to offset the effects of stressors, particularly in nursery pigs ultimately improving growth performance. However, the use of the antibiotics has been causing anxiety among consumers due to the fear of residues and the increasing microbial resistance which may affect the health of consumers (Yang et al. 2015; Valenzuela-Grijalva et al. 2017). Consequently, many developed countries have put in place restrictions on antibiotic use in animal production. For example, the European Union banned antibiotic use in animal production since 2006 (Bengtsson and Wierup 2006) while the U.S. Food and Drug Administration and Health Canada placed restrictions on antibiotic use in animals in 2017 with more countries expected to follow. A report on antimicrobial use and resistance in Africa found that MDR *E. coli* was 100% prevalent in South Africa, with isolates resistant to sulphonamides, tetracycline, and penicillin (Kimera et al. 2020). The high percentage of resistance to these antibiotics could be attributed to their widespread use, which is facilitated by their low cost and availability (Lekagul et al. 2019). Tetracycline resistance was found in high levels on pig farms in the Western Cape, South Africa (Ocloo et al. 2024). Tetracycline is widely used in South Africa’s livestock industry, accounting for 45% of antimicrobial sales in 2020 (National Deparment of Health 2021).

Nevertheless, the withdrawal of antibiotics use in animal feeds is not without its own challenges. It is therefore imperative to develop alternatives to the antibiotics with same effectiveness as the conventional antibiotics albeit being cost effective and sustainable. A variety of natural products, including phytogenic essential oils, organics acids, enzymes, probiotics and nanotechnology are being explored as potential alternatives to the conventional antibiotics growth promoters (Disetlhe et al. 2018; Yang et al. 2015; Valenzuela-Grijalva et al. 2017).

*Melia azedarach* is one of the medicinal plants that has potential as an alternative to antibiotics. The plant contains various phytochemicals including tri-terpenoids, flavonoids, and alkaloids with antimicrobial properties (Maciel et al. 2006; Ng et al. 2015). They also have antioxidant properties that can help with stress relief and improve performance in pigs. However, most phytochemicals have low in vivo bioavailability, efficacy, solubility, absorptive capacity, poor metabolism and delivery (Aqil et al. 2013). Encapsulation of phytochemicals onto nanoparticles is thus a modern technological advancement aimed at modifying phytochemical pharmacokinetics by improving their efficiency, stability, and solubility, as well as reducing their toxicity and target-site specificity (Huh and Kwon 2011). Zinc oxide nanocrystals are one of the utmost ordinarily produced nanoparticles. They have antimicrobial properties and are used as drug carriers in medicine. They’re also exploited in animal nutrition since they have good antimicrobial activity against *E. coli* and *Staphylococcus aureus* (Barreto et al. 2017). The nanoparticles may enhances gastric microbial diversity while improving gut morphology in weaner piglets. Ultimately, intestinal cross membrane transport system is potentiated and thus improving growth performance and antioxidant capacity (Zhao et al. 2014; Li et al. 2016).

The *M. azedarach* encapsulated of ZnO nanoparticles is therefore a potential effective alternative approach to the conventional antibiotics and need to be explored. As of right now, there is no marketable *Melia azedarach* seed based nano-ZnO being produced. This study, therefore, investigated if oral administration of green *M. azedarach* seed-based ZnO nanoparticles has no effect on growth performance, protein utilisation efficiency, haematology and nutritional status in pigs.

## Materials and methods

### Description of the study site

The research was conducted at the experimental farm (25º40.459’S, 26º10.563’E) of the North-West University, Mafikeng Campus (North-West province, South Africa). The farm is located at an altitude of 1226 m above sea level, with an annual rainfall of 450 mm. The ambient temperatures around the area range from 27 ºC to 37 ºC during summer and from 3 ºC to 25 ºC in winter.

### Synthesis of nanoparticles

Synthesis and characterization of nanoparticles were performed in the Department of Chemistry of North-West University, Mafikeng campus. Procedure can be looked up on my paper, “Biosynthesis of ZnO nanoparticles using *Melia azedarach* seed extract: Evaluation of the cytotoxic and antimicrobial potency, OpenNano 8 (2022) 10.1016/j.onano.2022.100068” (Dinga et al. 2022).

### Diet formulations and experimental design

A total of 48 Landrace 17–21 day old piglets were kept in well-aerated pens. The pens were furnished with nipple drinkers and feeding troughs. The pigs were assigned to 6 different treatments as follows: Negative Control (NC, No antibiotic), Treatment 2: Positive control (PC) given a conventional antibiotic (Oxytetracycline, 40 mg/kg feed); Treatment 3: Nano-ZnO 300 mg/L (N300ZnO), Treatment 4: Group given 150 mg/L *Melia azedarach* seed mediated nano-ZnO (N150MA), Treatment 5: Group given 300 mg/L *Melia azedarach* seed mediated nano-ZnO (N300MA), Treatment 6: Group given 450 mg/L *Melia azedarach* seed mediated nano-ZnO (N450MA). For each solution, the nanoparticles were dissolved in distilled water and kept refrigerated before use the next day. For Treatment 3 to Treatment 6 the nanoparticle solutions were administered by orally gavaging each piglet with 10 mL of each concentration of nanoparticles once daily in the morning (8:30 am) before feeding the animals. Oral administration of the *M. azedarach* seed based nano-ZnO solution every morning might have induced some stress from handling of the piglets which has the potential to affect the product effectiveness, however the *Melia azedarach* phytochemicals has antioxidant along with antimicrobial properties that might relieve stress and improve performance (Ahmed et al. 2012). The solution was administered to piglets per treatment group, starting with treatment 3 to treatment 6 each time. Each treatment was replicated 8 times. The study was conducted in a completely randomized design.

### Animal management

The pigs were housed in pens that meet the standard requirements for space and welfare. Appropriate enrichment methods were also applied. On introduction to the experimental pens, the pigs were treated against internal parasites before the initiation of the feeding trial with Ivomec. The pigs were permitted to adapt to dietary treatments as well as the surroundings for 14 days before the experiment. The experiment was conducted over a period of 49 days. The piglets were given feed and clean fresh water *ad libitum.* All protocols for the study were approved by the NWU-ANIMPROD Animal Research Ethics Committee of North-West University, in line with the National Research Council’s Guide for the Care and Use of Laboratory Animals and the Ethics number granted is NWU-00802-21-A5. The experimental pigs were in good health throughout the trial, no mortality or injury reported during the cause of the trial.

### Growth performance and cumulative weight gain

The animals were weighed at the beginning of the feeding trial to obtain the initial weight and weekly thereafter to obtain cumulative weight gain. The animals were provided with feed once in the morning and refusals were collected the following morning before feeding. Feed spillage was managed by choosing a feeder with a low waste design. By keeping the pig from backing out of the feeder to eat in an upright posture, feeders that enable pigs to eat while standing at the feeder will minimise waste. A feeder’s lip should be raised just enough to prevent spills, but no higher than eight inches (Schell et al. 2006). The difference between feed offered and refusals were used to determine the average daily feed intake (ADFI). Average daily gain (ADG) was calculated as follows:

$$ADG=\frac{W\left(T\right)-W\left(t_{0}\right)}{No.\:of\:days}$$

Where, t_0_ = initial time (days); T = final time; W(T) = final body weight (g), and W(t_0_) = initial body weight (g) of pigs. The total weight gain was obtained by subtracting initial weight from final weight at the end of the trial as follows: TWG = Final weight – Initial weight.

The feed conversion ratio was determined as the average daily feed intake and divided by the average daily weight gain.

$$\text{F}\text{C}\text{R}\:=\:\frac{Average\:daily\:feed\:intake}{Average\:daily\:gain}$$

### Protein utilisation efficiency

Protein consumed (PC g/pig) was calculated by multiplying the concentration of crude protein (CP_d_) in the diet (g/kg DM consumed) by feed intake over the feeding phase, whilst protein efficiency ratio (PER g/kg) was calculated by dividing mean body weight gain (BWG) by the mean protein consumed. Specific growth rate (SGR), which is percent growth per feeding phase, and growth efficiency (GE) were also calculated using the following formulas:

$$\text{P}\text{C}=FI\:\times\:CPd$$

$$\text{P}\text{E}\text{R}\:=\:\frac{\:BWG\:}{PC}$$

$$\text{S}\text{G}\text{R}\:=\:\frac{(In\:final\:weight\:-\:In\:\:initial\:weight)\:}{14\:d\:}\times\:100$$

$$\text{G}\text{E}\:=\:\frac{\:BWG\:}{Initial\:weight}$$

### Haematology and biochemistry

In each treatment at the end of the trial, approximately 4 mL of blood were obtained into 2 sets of vacutainer tubes (the red top for serum biochemistry, and the purple top for haematology). The samples for haematology were placed in a cooler box with ice packs and immediately sent for analysis, while the samples for biochemical parameters were held at room temperature then centrifuged at 2500 rpm for 15 min to get the serum, and then refrigerated at 4º C pending analysis. Haematological parameters were determined using an automated IDEXX LaserCyte Haematology Analyser (IDEXX Laboratories, Inc.). The biochemical parameters measured including Glucose, calcium, serum cholesterol, phosphorus, and triglycerides were analyzed using an automated IDEXX Vet Test Chemistry Analyser (IDEXX Laboratories Inc).

### Statistical analysis

Data on growth performance, protein utilisation efficiency, and blood parameters were analysed using GLM procedure of SAS (2010) with diet as the only fixed effect (model 1). Data on cumulative weight gain was measured every week and was analysed using mixed model procedure of SAS (2010) that took into consideration the effect of both diet and week of measurement (model 2) with diet as a fixed factor. The probability differences (PDIFF) option of SAS (2010) was used to perform pairwise comparisons of the least square means while contrasts (SAS 2010) were used to determine the specific effects of encapsulated nanoparticles and their interaction on different parameters.

The statistical models were as follows:

1 $$\text{Y}_{ij}={\mu}+\text{T}_{i}+{\varepsilon}_{ij}$$

Where: *Y*_*ij*_ = observation (growth parameters, PUE, and blood parameters), µ = population mean constant common to all observations, *T*_*i*_ = effect of diet, and ε_*ij*_ = random error term.

2 $$\text{Y}_{ijk}={\mu}+\text{T}_{i}+\text{W}_{j}+\left(\text{T}\:\text{*}\:\text{W}\right)_{ij}+{\varepsilon}_{ijk}$$

Where: *Y*_*ij*_ = observation (Cumulative weight gain parameter), µ = population mean constant common to the observation, *T*_*i*_ = effect of diet, T*W_*ij*_ = effect of nanoparticles interacting with week and ε_*ij*_ = random error term. For all tests, the level of significance was set at (*P* < 0.05).

## Results

### Growth performance

**Fig. 1:**
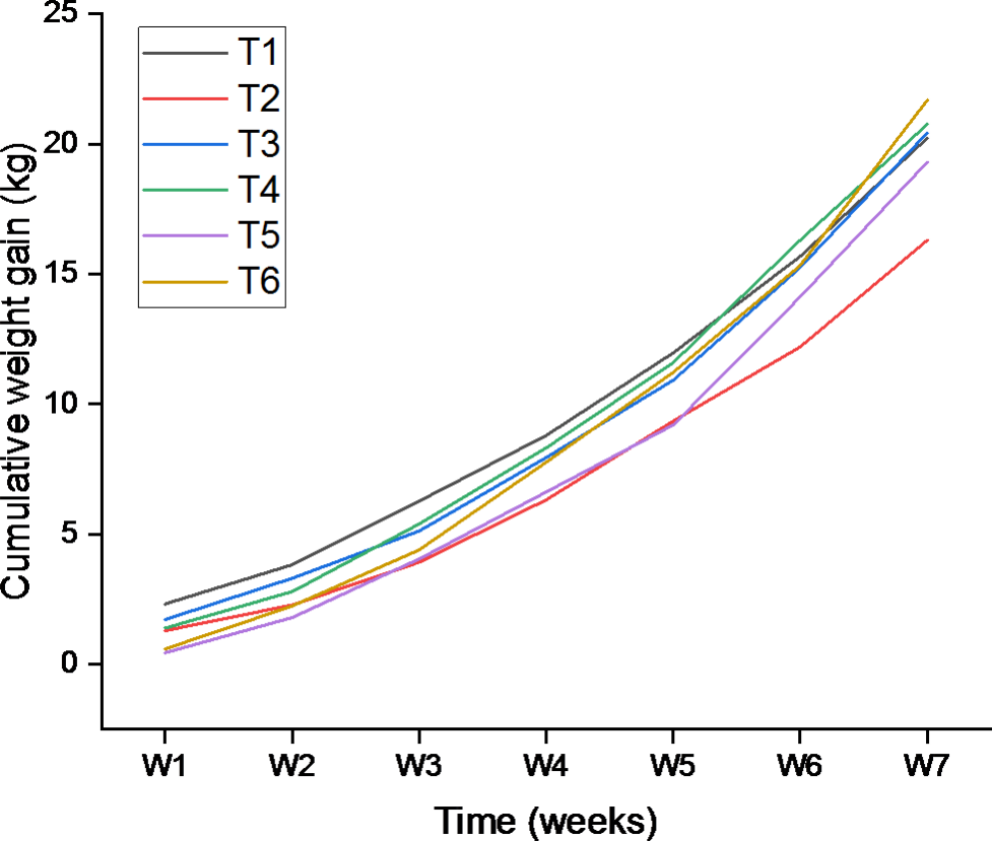
Effect of *M. azedarach* seed-mediated ZnO NPs on cumulative weight gain. NC- Treatment 1; PC- Treatment 2; N300ZnO- Treatment 3; N150MA- Treatment 4; N300MA- Treatment 5; N450- Treatment 6

Effects of *M. azedarach* seed-mediated nano-ZnO on growth performance are shown in Table 1. The results showed that *M. azedarach* seed-mediated ZnO nanoparticles had a significant effect on ADFI compared to the control groups. Figure 1 represents the cumulative weight gain of weaner pigs over the experimental period. A clear linear increase in weight gain was observed throughout the feeding period in all treatments. Among all treatments, the PC fed pigs had the lowest cumulative weight gain compared to all the other treatments, and those administered with *M. azedarach* seed-mediated nanoparticles had the highest gains.

**Table 1:** Effect of *M. Azedarach* seed-mediated ZnO nanoparticles on growth performance in weaner pigs

Parameters	Treatments							
	T1	T2	T3	T4	T5	T6	SEM	Significance
ADFI	0.742^a^	0.837^a^	0.901^b^	0.894^b^	0.862^ab^	0.888^ab^	0.25	***
ADG	0.407	0.332	0.416	0.423	0.389	0.447	0.03	NS
TWG	19.962	16.300	20.425	20.750	19.100	21.950	1.9	NS
FCR	2.010	2.666	2.219	2.229	2.380	2.111	0.2	NS

^a, b, c^ Means in the same row with different superscripts are significantly different (*P* < 0.05)

NC- Treatment 1; PC- Treatment 2; N300ZnO- Treatment 3; N150MA- Treatment 4; N300MA- Treatment 5; N450MA- Treatment 6

ADFI- Average daily feed intake; ADG- Average daily gain; TWG- Total weight gain; FCR- Feed conversion ratio

### Protein utilization efficiency

Table 2 shows the findings of protein utilization and growth efficiency in the pigs. Protein consumed and growth efficiency indices in weaner pigs were significantly impacted (*P* < 0.05) by *Melia azedarach* seed-mediated zinc oxide nanoparticles.

**Table 2:** Effect of orally gavaging *M. Azedarach* seed-mediated ZnO nanoparticles on protein utilisation efficiency in weaner pigs

Parameters	Treatment							
	T1	T2	T3	T4	T5	T6	SEM	Significance
PC	0.1187^a^	0.133^b^	0.144^c^	0.143^c^	0.138^bc^	0.142^c^	0.004	***
PER	162.480	120.600	141.175	143.608	136.505	152.654	10.911	NS
SGR	40.739	33.265	41.683	42.346	38.97	44.795	3.95	NS
GE	3.795^c^	2.506^a^	2.870^b^	2.780^ab^	2.463^a^	2.574^a^	0.3	*

^a, b, c^ Means in the same row with different superscripts are significantly different (*P* < 0.05)

NC- Treatment 1; PC- Treatment 2; N300ZnO- Treatment 3; N150MA- Treatment 4; N300MA- Treatment 5; N450MA- Treatment 6

PC- Protein consumed; PER-Protein efficiency ratio; SGR – Specific growth rate; GE- Growth efficiency

### Haematology

The whole blood parameters are presented in Table 3. As illustrated in the Table, the nanoparticles had a significant effect on eosin, platelet count, red blood cell distribution width, segmented neutrophils, and white blood cell count. Interestingly, all pigs orally administered with *M. azedarach* seed-mediated ZnO NPs had the lowest amount of WBC differential counts compared to control treatments. The nanoparticles had no significant effect on monocyte /lymphocyte, segmented neutrophil/lymphocyte apart from segmented neutrophils/monocyte ratios.

**Table 3:** Effect of green synthesized nanoparticles on haematology of weaner pigs

Parameters	Treatments							
	T1	T2	T3	T4	T5	T6	SEM	Sig
BASO (%)	0.6	0	0.3	0	0.3	0	0.23	NS
BASO ABS (x10^9/L)	0.2	0	0.07	0	0.1	0	0.07	NS
EOSIN (%)	0.6^a^	0.6^a^	3.0^c^	1.0^a^	2.3^b^	1.0^a^	0.4	**
EOSIN ABS (x10^9/L)	0.3^b^	0.14^a^	0.6^c^	0.19^a^	0.6^c^	0.2^a^	0.07	**
Hb (g/dL)	11.2	11.5	11.36	11.2	11.16	11.6	0.6	NS
LYMPH (%)	31.6	25.3	39	29.6	38	50.6	8.28	NS
LYMPH ABS (x10^9/L)	13.8	3.9	7.96	6.43	10.07	9.2	4.73	NS
MCHC (g/dL)	27.4	29.96	29.6	29.9	28.6	28.4	0.67	NS
MCV fL	56.2	60.86	57.06	58.6	60.1	61.5	1.69	NS
MONO%	3.3	7.3	9.6	8	8.3	4.33	1.75	NS
MONO ABS x10^9/L	1.4	1.35	2.02	1.76	1.95	0.78	0.38	NS
PCV%	38.5	39.3	38.7	37.5	38.8	44.2	2.24	NS
PLAT x10^9/L	276^b^	242.3^b^	275^b^	467^c^	172.3^a^	447^c^	47.69	**
RBC x10^12/L	6.7	6.5	6.74	6.3	6.46	7.26	0.23	NS
RDW%	26^c^	20.1^b^	20.2^b^	17.3^a^	20.1^b^	20.3^b^	1.01	**
SNEUT%	71.3	61.6	48	61.6	52	39.6	9.29	NS
SNEUT ABS x10^9/L	25.6^c^	11.4^b^	9.4^a^	13.6^b^	14.6^b^	6.81^a^	2.48	**
WBC x10^9/L	40.5^c^	17.0^a^	20.1^b^	22.05^b^	23.3^b^	17.39^a^	4.49	*
MONO /LYMPH	0.3	0.3	0.27	0.27	0.23	0.08	0.1	NS
SNEU/LYMPH	9.9	3	1.38	2.1	1.56	0.74	2.74	NS
SNEU/MON	22.6^c^	8.8^b^	5.9^a^	7.75^a^	7.47^a^	8.73^b^	3.42	*

^a, b, c^ Means in the same row with different superscripts are significantly different (*P* < 0.05)

Baso- Basophils; Lymph- Lymphocytes; Eosin- Eosinophils; Mono- Monocytes; RBC- Red blood cell count; MCHC- Mean cell haem.cons; RDW- Red cell distribution width; RBC- Red blood cell count; PCV- Haematocrit; SNEUT- Segmented Neutrophils; PLAT- Platelet count

NC- Treatment 1; PC- Treatment 2; N300ZnO- Treatment 3; N150MA- Treatment 4; N300MA- Treatment 5; N450MA- Treatment 6

### Nutritional status

Table 4 shows the effect of *M. azedarach* seed-mediated ZnO NPs on serum biochemistry. There was no effect of treatment on serum between calcium levels and they were all within the normal range. However, phosphorus levels were all above normal levels and were statistically different. There was no effect of treatment on nutritional enzymes among all the treatments. In addition, there was no effect of treatment in kidney enzymes.

**Table 4:** Effect of *M. Azedarach* seed-mediated ZnO NPs in serum biochemistry in weaner pigs

Parameters	Treatment							
	T1	T2	T3	T4	T5	T6	SEM	Sig
CA (mmol/L)	2.9	2.6	2.6	2.7	2.7	2.83	0.09	NS
PHOS (mmol/L)	5.2^c^	4.5^b^	4.1^a^	3.9^a^	4.3^a^	4.75^b^	0.25	*
UREA(mmol/L)	2.3	2.3	2.6	2.7	3.4	2.97	0.49	NS
CREA (µmol/L)	86	77.6	88.3	88.3	99	85	8.06	NS
GLU (mmol/L)	4.1	2.7	3.15	3.6	4.2	4.2	0.43	NS
CHOL (mmol/L)	2.5	2.3	2.8	2.7	3.16	2.76	0.19	NS
TRIG (mmol/L)	0.45	0.4	0.6	0.5	0.7	0.4	0.07	NS

^a, b, c^ Means in the same row with different superscripts are significantly different (*P* < 0.05)

CA- Calcium; PHOS- phosphorus; UREA- Urea; CREA- Creatine; GLU- Glucose; CHOL- Cholesterol; TRIG- Triglycerides

NC- Treatment 1; PC- Treatment 2; N300ZnO- Treatment 3; N150MA- Treatment 4; N300MA- Treatment 5; N450MA- Treatment 6

## Discussion

In pig production systems, antibiotic growth promoters such as ZnO are commonly given to piglets to address post-weaning growth challenges. Zinc is a trace element that constitutes the important components of many metallo-enzymes and digestive enzymes. It plays a critical role in growth, immune function, and nutrient metabolism in animals (Baker and Ammerman 1995; Wang et al. 2009). Encapsulation of zinc nanoparticles has the potential to improve the effectiveness of ZnO. In the current study *Melia azedarach* seed-mediated ZnO NPs were used as an alternative to antibiotics in pigs. From the results, pigs given *M. azedarach* seed-based ZnO NPs had higher ADFI, compared to the control groups. This could be because the *M. azedarach* phytochemicals that coated the nanoparticles had the effect of potentiating the bioavailability uptake of the ZnO nanoparticles (Delie 1998), resulting in improved growth performance and cumulative weight gain with T6 (N450MA) having the highest gains when compared to conventional antibiotics (PC-oxytetracycline and NC-without antibiotic). Moreover, some of the phytochemicals are bioactive and hence have potent antioxidant properties that could have influenced cell growth (Ahmed et al. 2012). Many factors, such as size, capping agent nature (plant) and coating may influence NP uptake by cells.

Protein consumption has been reported to be linearly proportional to feed intake until it reaches a maximal plateau (Whittemore and Fawcett 1976; Campbell et al. 1985). Pigs with high feed intakes reach the plateau early in life, and daily nitrogen retention remains constant over a wide range of live weights after that (Tullis 1982). Many factors influence the efficient utilization of dietary protein, including the animal’s physiological requirements for amino acids, genetic capacity for protein deposition, dietary amino acid digestion/absorption, antibiotic use, stress and metabolism/partitioning of ingested amino acids (Kim and Pluske 2016). Orally administered *M. azedarach* seed-mediated ZnO NPs had a positive effect on the protein consumed and specific growth rate when compared to the control groups. This could be due to increased peptic activity and increased N digestibility via gut pH changes (Ragaa and Korany 2016) stimulated by the *M. azedarach* extracts. Drug dissolution and solubility, its release, stability and intestinal permeability are all affected by gastrointestinal (GI) pH. It is therefore surmised that *M. azedarach* phytochemicals could have significantly altered gut pH, resulting in substantial effects on Zinc nanoparticle absorption and bioavailability, ultimately improving growth efficiency in pigs as previously reported in the study by Xu et al. (2013).

Haematological analysis is often critical in the assessment of the health and general welfare of animals. Haematological parameters are critical for assessing ZnO-NP toxicity (Yang et al. 2017). According to Dobrovolskaia et al. (2016), nanoparticles have the potential to cause immunotoxicity because they disrupt the immune system. It has been demonstrated that direct toxicity or an immune-mediated drug injury results in a decrease in the neutrophil count of pigs treated with *M. azedarach* seed mediated ZnO NPs (Arika et al. 2016). Due to the structural bending of red blood cells caused by the metal, there is a decrease in the number of red blood cells (RBCs), which in turn results in a decrease in the levels of haemoglobin in the blood seen in T4 (N150MA) and T5 (N300MA) (Venkatachalam and Natarajan 2014). In contrast to control groups, the ZnO NP-treated pigs had higher MCHC and lymphocyte counts, which is inconsistent with the findings of Shaban et al. (2021).

Leukocytes are important components of the body’s defense system and can be indicative of an infectious processes in general. The high WBC count, neutrophils, neutrophil/monocytes ratio in the NC group was expected and could be indicative of the strain on the immune system in the absence of antibiotics or their alternatives. In the pigs given the *M. azedarach* seed-mediated ZnO NPs the phytochemicals reduced the stress-related leukocytosis (Dubreuil and Lapierre 1997; Casas-Diaz et al. 2015). White blood cell ratios could be employed as valid biomarkers for any inflammation triggered by feed-induced stress. Compounds of a similar sort have been found to diminish the haematological problems linked with aflatoxins and mycotoxins in feeds (Abdel-Wahhab and Aly 2005). Overall, red blood cell indices were found to be within expected values for healthy pigs (Perri et al. 2016).

Across all the treatment groups there was no effect of nano-ZnO administration on minerals observed. Observations have been made in some studies that supplementation of a basal diet with Zn negatively affected the absorption and blood concentration of Phosphorus (Phiri et al. 2009) and some bivalent metals including calcium (Suttle 2010; Goff 2018). The lack of effect for the nano-ZnO administered in the current study could be due to the low levels of supplementation given and hence the non-significant effect on the metabolism of other minerals as observed in other studies (Phiri et al. 2009; Goff 2018; Hosseini-Vardanjania et al. 2020).

The most often used measure for renal function is serum creatinine, although it is neither sensitive nor specific for early identification of kidney injury. Creatinine is a chemical waste produced during muscle metabolism. It travels through the bloodstream to the kidneys, which filter out the majority of the creatinine and excrete it with urine. Thus, an elevated creatinine level, as seen in the results, indicates impaired kidney function as a result of exposure to zinc oxide nanoparticles. Metal nanoparticles are known to inhibit cell activity by mimicking epigenetic changes (Ezealisiji et al. 2019; Guanalan et al. 2012; Vanaja and Annadurai, 2012). Alkaladi et al. (2015) discovered that O. niloticus has a higher rate of creatinine fixation using a renal working test. The results of this discovery were comparable to the results of the current investigation.

The measurement of serum uric acid aids in assessing the efficiency of the body’s uric acid production and elimination. Normally, uric acid dissolves in blood, passes through the kidneys, and is then eliminated in urine. Based on the study, it can be concluded that the concentration of green synthesised ZnO nanoparticles increased along with the serum uric acid level. Although Ezealisiji et al. (2019) found that urea levels decreased in a time- and dose-dependent manner, suggesting that repeated exposure to the zinc oxide nanoparticles could lead to oxidative stress since uric acid is an anti-oxidant, Elghobashy et al. (2001) explained this rise in metal toxicity, which results in neurotic changes in the kidney’s glomerulus filtration repeat.

It can also be inferred that, albeit not very significantly, being exposed to zinc oxide nanoparticles raises total cholesterol. This results from harm to the liver cells that manufacture and eliminate cholesterol from the body, and over time, this may cause certain abnormalities in the cardiovascular system (Ezealisiji et al. 2019). Urea levels rise when renal function declines. In the current study, there was no significant effect of nano-ZnO administration on creatinine and urea levels, indicating the absence of renal dysfunction or zinc oxide nanoparticle toxicity in the weaner pigs. These findings are consistent with those previously published by Belewu et al. (2021). The toxicity of nano-zinc oxide is associated with the dose and duration of the experimentation on the animals (Swain et al. 2016).

In comparison to the control groups, *Melia azedarach* seed-mediated zinc oxide nanoparticles did not affect cholesterol, triglycerides, or glucose levels. High cholesterol levels could be linked to a metabolic response in fasting pigs to unmet energy needs, which results in increased lipid mobilization and gluconeogenesis activity. Fasting causes a negative energy balance, and triacylglycerol supplies free fatty acids for oxidation, which can then be converted to ketone and acetone, which serve as a substitute for glucose (Gupta and Houston 2017). ZnO NPs improve the release and uptake of hepatocytes’ glucose (Brown 2000). It is noteworthy that zinc acts as an insulin-like agent since it gets involved in many critical enzymatic systems, which take part in insulin manufacturing, storing, secretion, carbohydrates metabolism and antioxidative activities (Miao et al. 2013). The current study found that serum glucose levels were comparable among experimental groups, which was consistent with previous findings (Belewu et al. 2021).

## Conclusion

The current study findings show that oral administration of *M. azedarach* seed-mediated ZnO nanoparticles improved growth performance and cumulative weight gain when compared to conventional antibiotics. Nanoparticles are effective because they are soluble, bioavailable and have a higher cellular uptake. When compared to oxytetracycline, nanoparticles had a greater improvement on average daily feed intake, protein consumed, growth efficiency, haematology (EOSIN(%), EOSINABS(x10^9/L), PLATx10^9/L, RDW%, SNEUTABSx10^9/L, WBCx10^9/L and SNEU/MON) and serum phosphorus levels in pigs. These findings demonstrate that nanoparticles encapsulated in phytochemicals are a potent alternative to conventional antibiotics and can be used in pig production systems without detectable negative effects on general health and performance.

## Data Availability

Not applicable.
